# Energy Efficiency of Task Allocation for Embedded JPEG Systems

**DOI:** 10.1155/2014/718348

**Published:** 2014-04-10

**Authors:** Yang-Hsin Fan, Jan-Ou Wu, San-Fu Wang

**Affiliations:** ^1^Department of Computer Science and Information Engineering, National Taitung University, Taitung 95002, Taiwan; ^2^Department of Electronic Engineering, De Lin Institute of Technology, New Taipei 23654, Taiwan; ^3^Department of Electronic Engineering, Ming Chi University of Technology, New Taipei 24301, Taiwan

## Abstract

Embedded system works everywhere for repeatedly performing a few particular functionalities. Well-known products include consumer electronics, smart home applications, and telematics device, and so forth. Recently, developing methodology of embedded systems is applied to conduct the design of cloud embedded system resulting in the applications of embedded system being more diverse. However, the more energy consumes result from the more embedded system works. This study presents hyperrectangle technology (HT) to embedded system for obtaining energy saving. The HT adopts drift effect to construct embedded systems with more hardware circuits than software components or vice versa. It can fast construct embedded system with a set of hardware circuits and software components. Moreover, it has a great benefit to fast explore energy consumption for various embedded systems. The effects are presented by assessing a JPEG benchmarks. Experimental results demonstrate that the HT, respectively, achieves the energy saving by 29.84%, 2.07%, and 68.80% on average to GA, GHO, and Lin.

## 1. Introduction

Smarter, smaller, and portable characteristics make embedded systems to serve diverse functionalities. Nowadays, embedded systems have rapidly increasing requirements in the applications as in automobiles, avionics, and mobile devices. According to the IDC [1] reports, nearly 1 billion smart connected devices were shipped in 2011 and it would be double by 2016. Likewise, the Intel [2] predicts that there will be more than 30 billion devices constantly linked and another 150 billion fitfully connected in the end of this decade. It will greatly increase the demand of energy while these devices are served. However, the more embedded systems that are served, the more energy they consume. As a result, Bernd [3] summarizes the market and technology analysis which are towards energy efficiency for mobile devices, cloud computing, and storage services.

From an architectural perspective, all embedded systems have hardware and software components. These hardware components, such as* application specific integrated circuits* (ASIC) or standard logic, offer specific functionalities or programmable gates when developing hardware circuits. Conversely, the software components, such as microcontrollers or programmable* digital signal processing* (DSPs), provide an environment for various application programs. By assembling these programmable hardware and software, any embedded system can be developed depending on system specifications.

Inside the embedded systems there are a few tasks that are designed by programmable hardware or software components. Each task consumes energy regardless of the forms in either programmable hardware or software components. Energy consumption is classified as dynamic and static energy consumption based on its mode of state. The dynamic energy consumption is defined while the task is working for providing functionalities. On the other hand, task consumes static energy dissipation when its state is idle. For an embedded system with *n* tasks, executing one task consumes dynamic energy and the other tasks arise static energy consumption. For example, when task 1 runs, task 2, task 3 till task *n* occur static energy consumption. In consideration of task 2 runs, it consumes dynamic energy consumption, at the same time, task 1, task 3, task 4 till task *n* occur static energy consumption. To iterate the process for every task execution, the energy consumption of embedded system can be assessed.

The aforementioned statements manifest embedded systems to incessantly consume energy for repeatedly performing a few particular tasks. In order to improve energy consumption, this study proposes* hyperrectangle technology* (HT) to embedded system target to obtain energy saving. This paper is organized as five sections. In Section 2, we investigate some previous works with respect to energy saving for embedded system.  Section 3 describes HT for embedded systems to achieve energy saving.  Section 4 demonstrates experimental results of* joint photographic experts group* (JPEG) encoding system that is performed by HT. We conclude this work in Section 5.

## 2. Preliminary Works

Researchers pay much attention to energy consumption of embedded systems with respect to the fields of processor energy consumption, real-time power consumption, dynamic power consumption, and scheduling power consumption. From the processors viewpoints, Vîlcu [4] aims at real time embedded system to minimize the CPU power consumption. First, he studies task execution in the power consumption of processor(s). Then, he finds the effects of optimal configuration processor(s) for energy consumption. Finally, he defines globally optimal scheduling which gains minimal energy consumption for homogeneous multiprocessor system. Gao et al. [5] present* energy-efficient architecture for embedded software* (EAES) and dynamic energy-saving method to solve energy-saving problem. The former uses a processor with dynamic voltage scaling capability, FPGA modules, and extends directed acyclic graph to embedded system. The latter adopts preassignment to achieve dynamic runtime scheduling and minimize energy consumption. Qiu et al. [6] discuss the execution time of tasks with conditional instructions or operations problem. They adopt probabilistic random variable approach to model execution time of tasks. Then, they propose practical algorithm VACP to minimize energy consumption for uniprocessor embedded systems. Silva-Filho and Lima [7] state that memory hierarchy consumes power up to 50% in microprocessor system. Consequently, they propose an automated architecture exploration mechanism to NIOS II processor and memory hierarchy with parameter variation. The experimental results show the reduction of energy consumption is approximately 27%. In 2008, Zeng et al. [8] present generalized* dynamic energy performance scaling* (DEPS) framework to hard real-time embedded systems for exploring application-specific energy-saving potential issue. Three energy performance tradeoff technologies, DHRC, DVFS, and DPM, are integrated into DEPS. Their experimental results of simulation show the static DEPS has been improved, respectively, 13.6% and 13.7% in DVFS and DHRC. Also, dynamic DEPS improves 5.7% when comparing to static DEPS.

Real-time power information is a valuable data for software designer for battery-powered embedded systems. Genser et al. [9] propose power profiling approach to collect real-time power information at early designing stages. Moreover, they present an emulation-based power profiling approach to achieve real-time power analysis for embedded systems. Because the power information is collected at early designing stages, the development efficiency and time to market are improved. In 2008, Elewi et al. [10] first discuss the real-time scheduling of dependent tasks problem and then present enhanced* multispeed* (MS) algorithm for energy saving. With energy consumption problem of battery-powered embedded systems, Casares et al. [11] aim at embedded smart camera to analyze the power consumption and performance. Not only graph of energy consumption but also instructions of collections are presented. They conclude the importance of lightweight algorithm, the time of transfer data, and transferred data type.

Dynamic power consumption of* field programmable gate array* (FPGA) is discussed in [12, 13]. In 2009, Tsang and So [12] adopt precomputation approach to reduce dynamic power consumption in commercial off-the-shelf FPGAs. The experimental results of comparator show that 83% of dynamic power in logic or 43.1% of total dynamic power is reduced if the increased resource consumption can be negligible. In 2010, Bhandari et al. [13] present fly partial reconfiguration as well as scaling the clock on FPGA for reducing dynamic power consumption in embedded system. They conclude that the factors of dynamic power dissipation consist of application, architecture, and reconfigurable time.

The application of different algorithms to arrange scheduling issues for reducing power consumption is discussed in [14, 15]. In 2010, Bashiri and Miremadi [14] investigate* earliest-deadline-first* (EDF) and* rate-monotonic* (RM) algorithms on power efficiency of task scheduling. The results show that the BF-EDF and FF-EDF have the best power efficiency. In 2011, Cho et al. [15] propose power-saving scheduling algorithm and use soft-deadline to reduce energy consumption by about 40%. However, it is a trade off on performance and power savings for embedded systems. Kan et al. [16] present a heuristic algorithm called TGPM-ALL with interior point method to handle the frequency assignment on multiple soft-deadlines embedded systems. Their empirical results show the effectiveness in comparing TGPM-ALL with TGPM-1 and BEST algorithms.

## 3. Hyperrectangle Approach

*Task graph* (TG) is a conceptual graph that facilitates to describe operation for embedded system. TG comprises of vertices (*V*), edges (*A*), and levels (*L*) that can be represented by a 3-tuple set, *G*(*V*, *A*, *L*). The *V* is a unit of work which may take dependencies one or more antecedents. The *A* is used to exhibit the flow among *V*. The *L* indicates the order of works for *V*. Based on TG, we propose* energy-consumed task graph* (ETG) as system model of HT that adds a factor of energy consumption on TG.

### 3.1. System Model

**Figure 1:**
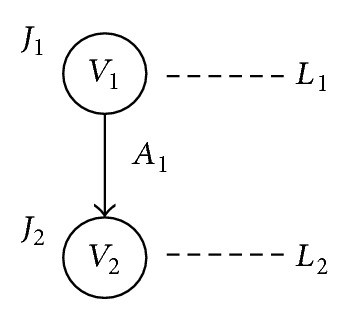
A simple ETG with two tasks.

ETG comprises of vertices (*V*), edges (*A*), levels (*L*), and energy consumption (*J*) that can be represented by a 4-tuple set, *G*(*V*, *A*, *L*, *J*). Symbol *V* stands for task that is a working unit on embedded system. For example, one task is represented as *V*_1_ and a number of tasks are labeled as *V*_2_, *V*_3_, and *V*_4_, and so forth. Hence, application program inside embedded system can be defined as a set of tasks as *V*_1_, *V*_2_, and *V*_3_ to *V*_*n*_. Another symbol *A* is used to direct work flow of applications among *V*s. For instance, symbol *A*_1_ guides the working flow from task *V*_1_ to another task *V*_2_. Moreover, two tasks are connected by *A*_1_ that implies their location on different levels. Label *L* defines the state of *V* and the height of ETG. The state is organized into two categories. One state is named working (*W*) and the other idle (*I*). Both states simultaneously incur when a symbol *A*_1_ activates. For instance, working state *W*_1_ on *V*_2_ and idle state *I*_1_ on *V*_1_ separately form when *A*_1_ activates. Sign *J* denotes the energy consumption of *V*. Each task consumes energy depending on either *W* or *I*.  Figure 1 displays a ETG with 2 vertices (i.e., *V*_1_ and *V*_2_), 1 edge, 2 energy consumption (i.e., *J*_1_ and *J*_2_), and 2 levels (i.e., *L*_1_ and *L*_2_).

### 3.2. Energy Consumption Definition

Power consumption of the task is classified into dynamic or static power consumption according to their state of work. Dynamic power consumption *D* occurs while the task locates *W*. On the contrary, the task in *I* consumes static power consumption *S*. Take Figure 1 as an example, the *V*_1_ and *V*_2_ first consume *D* and *S* separately in *L*_1_ because the former locates at *W* and the latter places on *I*. After that, the *A*_1_ directs the work flow to *L*_2_. In *L*_2_, the *V*_2_ and *V*_1_ individually consume *D* on *W* and *S* on *I*. In summary, both *D* and *S* are consumed by *V* depending on *W* or *I*. It should be noticed that each task must consume energy at any time even though it is idle.

Low power dissipation model and analysis for embedded systems are discussed by Fan et al. [17]. They derive power dissipation with dynamic and static power dissipation from TG. The expression of the sum of power consumption for embedded system is calculated by using

(1) P=(L−1)×(Ps,t1+Ps,t2+⋯+Ps,tn)+(Pd,t1+Pd,t2+⋯+Pd,tn),

where *L* is the height of TG, *P*_*s*_ is static power consumption, *P*_*d*_ is dynamic power consumption, and *t*_1_, *t*_2_,…, *t*_*n*_ is a set of task. In consideration of energy consumption, the energy consumption is formulated as follows:

(2) $$\begin{array}{c} E=P\times T, \end{array}$$

where *P* is power dissipation and *T* represents execution time. Owing to power consumption which is divided into *D* and *S*, the energy consumption is categorized into dynamic (*E*_*d*_) and static (*E*_*s*_) energy dissipation. Moreover, each task can be separately implemented as two forms of *f* as* hardware circuit* (HC) and* software component* (SC) so that the energy consumption of embedded system can be formulated as follows:

(3) $$\begin{array}{c} E_{d,v_{i}}^{f}=P_{d,v_{i}}^{f}\times T_{d,v_{i}}^{f},\\ E_{s,v_{i}}^{f}=P_{s,v_{i}}^{f}\times T_{s,v_{i}}^{f}, \end{array}$$

where *d* is dynamic energy consumption, *s* is static energy consumption, *f* is a form of hardware circuit or software component, and *v* is task, *i* = 1,2,…, *n*. In summarizing equations from (1) to (3), the total energy consumption can be derived as follows:

(4) E=(L−1)×(Es,v1f+Es,v2f+⋯+Es,vnf)+(Ed,v1f+Ed,v2f+⋯+Ed,vnf),

where *L* is the height of ETG, *E*_*s*_ is static energy consumption, and *E*_*d*_ is dynamic energy consumption.

### 3.3. Hyperrectangle Model

To construct hyperrectangle model of energy consumption for embedded system, we first analyze the energy consumption of ETG with two tasks which is shown in Figure 1. Then derive complicated model from it. By holding the principle of one task that has two forms (i.e., HC and SC), a ETG with two tasks (i.e., *v*_1_ and *v*_2_) can be constructed four embedded systems namely HC-HC (*E*_1_), HC-SC (*E*_2_), SC-HC (*E*_3_), and SC-SC (*E*_4_). According to (3), the energy consumption of *E*_1_, *E*_2_, *E*_3_, and *E*_4_ can be defined in the following:

(5) E1=Ed,v1HC+Es,v2HC+Ed,v2HC+Es,v1HC,

(6) E2=Ed,v1HC+Es,v2SC+Ed,v2SC+Es,v1HC,

(7) E3=Ed,v1SC+Es,v2HC+Ed,v2HC+Es,v1SC,

(8) E4=Ed,v1SC+Es,v2SC+Ed,v2SC+Es,v1SC.

The first and the second terms or the third and the fourth terms from (5) to (8) prove the description in Section 3.1, which indicates that *W* and *I* simultaneously incur while a symbol *A*_1_ activates. Moreover, the third and the fourth terms can be regarded as mutual functions as the second and the first terms. Consequently, the third and the fourth terms from (5) to (8) can be formulated as the third term in the following:

(9) E1=Ed,v1HC+Es,v2HC+f(Ed,v1HC,Es,v2HC),

(10) E2=Ed,v1HC+Es,v2SC+f(Ed,v1HC,Es,v2SC),

(11) E3=Ed,v1SC+Es,v2HC+f(Ed,v1SC,Es,v2HC),

(12) E4=Ed,v1SC+Es,v2SC+f(Ed,v1SC,Es,v2SC).

From the axial coordination's perspective, the first and the second terms from (9) to (12) can be represented as four points of a rectangle *R* ^1^. Moreover, the third term from (9) to (12) forms four points in another rectangle *R* ^2^ in the tridimensionality. As a result, the hyperrectangle model of energy consumption for embedded system with two tasks is constructed and transferred to the three-dimensional space.

Based on the previous description, the hyperrectangle model of energy consumption for embedded system with two tasks can be defined in the following.

For a rectangle *R* ^2^, it has vertex (*x*_*i*_, *y*_*j*_), *i*, *j* = 1,2, and a solution set *D*⊆*R* ^2^. A given function *f* : *D* → *R*, *f*(*x*, *y*) = *u* and *b* ∈ *R*, the solution can be obtained as follows:

(13) $$\begin{array}{c} x_{i}+y_{j}+f\left(x_{i},y_{j}\right)≦b,\;\text{where}\;i,j=1,2. \end{array}$$

**Example 1**

**Figure 2:**
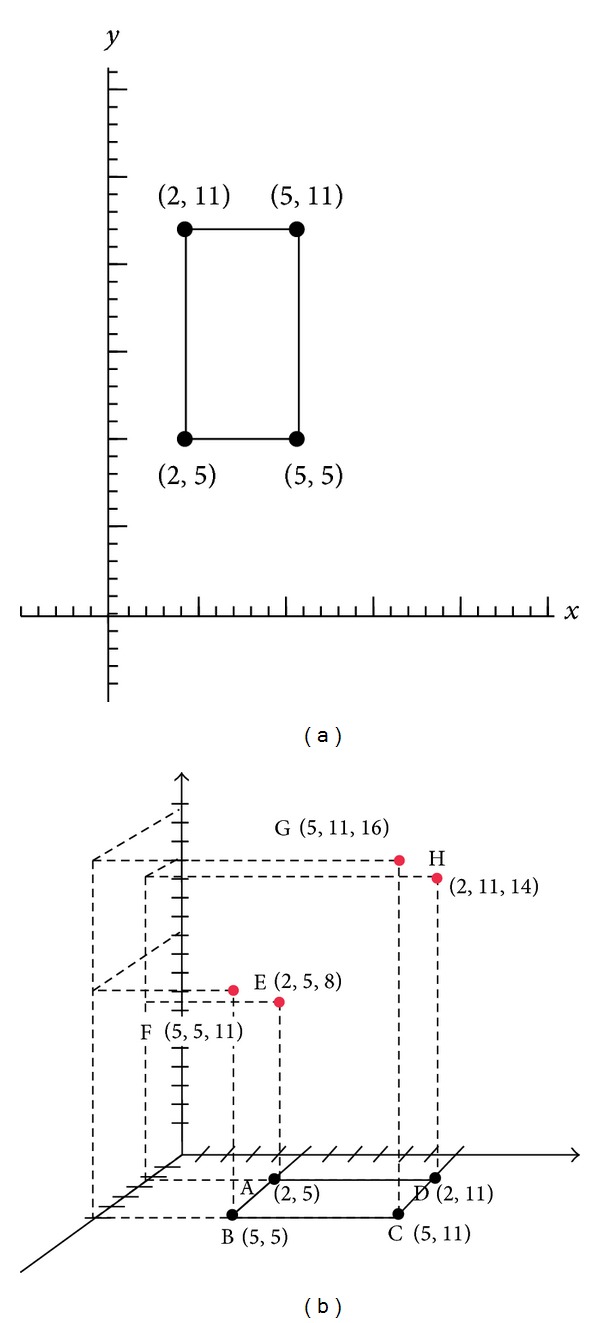
Example of a rectangle *R* ^2^ in hyperrectangle model.

Figure 2(a) shows a set of vertex *D* = {(2,5), (5,5), (2,11), (5,11)}.  Figure 2(b) displays *f* : *D* → *R* where *f*(2,5) = 8, *f*(5,5) = 11, *f*(2,11) = 14, and *f*(5,11) = 16. For a given *b* = 20, the solution comprises {(2,5), (5,5)} since it meets *x*_*i*_ + *y*_*j*_ + *f*(*x*_*i*_, *y*_*j*_)≦*b*.

**Example 2**

**Figure 3:**
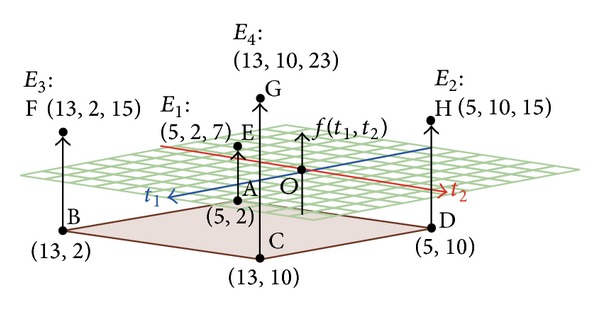
Hyperrectangle schema of embedded system with two tasks.

Table 1 displays an energy consumption example of embedded system with two tasks. The evaluating factors of energy consumption include the name of task, dynamic, and static energy consumption of HC and SC.  Figure 3 shows a hyperrectangle schema of embedded system with two tasks (i.e., *v*_1_ and *v*_2_). According to (9) to (12), the first and the second term are transferred to *XY* axes for *E*_1_, *E*_2_, *E*_3_, and *E*_4_, where locates at points A, D, B, and C. Points E, H, F, and G are marked for the third term from (9) to (12). We observe the most energy consumption occurring at point G (i.e., 13 + 10 + 23 = 46), which comprises SC-SC (*E*_4_). On the other hand, the HC-HC (*E*_1_) consumes the fewest energy consumption. Eventually, the energy consumption can be improved if one task is substituted from SC to HC where less energy is consumed.

Similarly, an embedded system with three tasks can be defined as follows. For a cuboid *R* ^3^, it has vertex (*x*_*i*_, *y*_*j*_, *z*_*k*_), *i*, *j*, *k* = 1,2, and a solution set *D*⊆*R* ^3^. A given function *f* : *D* → *R*, *f*(*x*, *y*, *z*) = *v* and *b* ∈ *R*, the solution can be obtained from the following:

(14) $$\begin{array}{c} x_{i}+y_{j}+z_{k}+f\left(x_{i},y_{j},z_{k}\right)≦b,\;\text{where}\;i,j,k=1,2. \end{array}$$

According to (13) and (14), we summarize the general expression for embedded system with *n* tasks as follows. For a hyperrectangle *R* ^*n*^, it has vertex, (*x*_(1,*i*_1_)_, *x*_(2,*i*_2_)_,…, *x*_(*n*,*i*_*n*_)_), *i*_*j*_ = 1,2, and a solution set *D*⊆*R* ^*n*^ where *D* is shown in (15). Given a function *f* : *D* → *R* and *b* ∈ *R*, the solution can be obtained as follows:

(15) $$\begin{array}{c} D=\left\{\left(x_{\left(1,i_{1}\right)},x_{\left(2,i_{2}\right)},\ldots ,x_{\left(n,i_{n}\right)}\right)\;|\;i_{j}=1,2\right\}, \end{array}$$

(16) $$\begin{array}{c} x_{\left(1,i_{1}\right)}+x_{\left(2,i_{2}\right)}+\cdots +x_{\left(n,i_{n}\right)}+f\left(x_{\left(1,i_{1}\right)},x_{\left(2,i_{2}\right)},\ldots ,x_{\left(n,i_{n}\right)}\right)\leq b, \end{array}$$

where 1≦*j*≦*n*.

Applying hyperrectangle approach to embedded systems for gaining energy consumption consists of the following steps. First, the number of tasks *U* of embedded system must be defined. Next, separately constructing the number of tasks *U*_hc,*i*_ with HC and *U*_sc,*j*_ with SC being the same as *U*. Third, constructing sets of *f*(*U*_hc,*i*_) and *f*(*U*_sc,*j*_) depicts energy consumption for each task. Therefore, energy consumption of each task can be fast evaluated. Fourth, constructing the first embedded system ES_a_ that comprises a set of tasks with *U*_sc_. Fifth, the task with the most energy consumption in *f*(*U*_sc,a_) is swapped with *f*(*U*_hc,a_). After the swapping process is iterated until each task is made of *U*_hc_, the HT exploits a set of embedded systems HT_x_ according to the number of *U*_hc_ and *U*_sc_. The first embedded system ES_1_ is assembled by one of two *U*_hc_ and *U*_sc_. If *U* is odd, extra SC is set preceding privilege to deploy to the ES_1_. The second embedded system ES_2_ is set where the number of tasks with SC is more than HC. Alternatively, the number of tasks with HC that is more than SC is set to the third embedded system ES_3_. In setting ES_2_ and ES_3_ until ES_m_, the above process is executed repeatedly. We observe that ES_2*i*_ and ES_(2*i*+1)_ form the drift effect with SC and HC, respectively.

## 4. Experimental Results and Analysis

**Figure 4:**
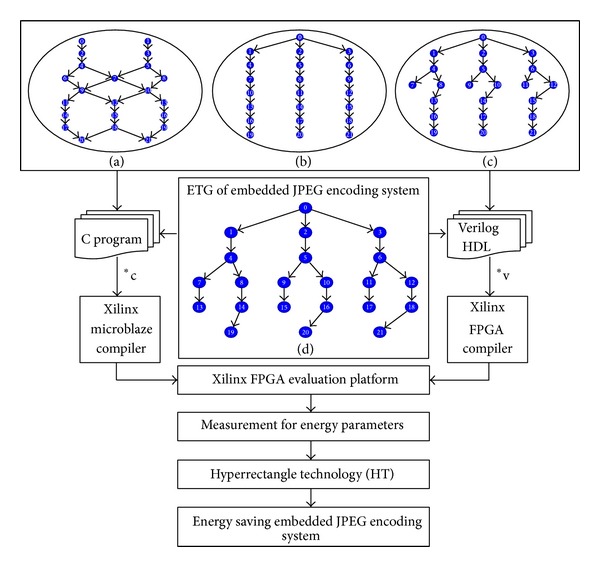
Flow chart of experimental setup.

The experimental platform is Xilinx FPGA ML507 [18].  Table 2 presents the technology of system parameters. The tested example is* joint photographic experts group* (JPEG) encoding system that consists of 22 tasks and 9 levels. From level 1 to 9 in Figure 4(a), the number of tasks is 2, 2, 2, 3, 2, 3, 3, 3, and 2, respectively. Each task is individually implemented as hardware circuit and software component form, which are designed by Verilog programming language and C programming language. Figure 4 demonstrates the flow chart of experimental setup.

The measured data of energy consumption is shown in Table 3. In the Task column, it shows the name of task that works in the JPEG encoding system. For the dynamic and static energy consumption of hardware circuit, it is displayed in column 2 and 3. The software tasks with dynamic and static energy consumption are illustrated in column 4 and 5.

Table 4 lists the experimental results of the proposed approach. Embedded systems column displays the results according to (16). In the energy dissipation column, it is calculated via (4). The ES_l_ is set to ES_1_, ES_k_, and ES_m_ is set to ES_2_ and ES_3_, respectively. On one hand, the SC drift effect, respectively, diffuses from ES_k_ to ES_a_. Similarly, the HC drift effect diffuses from ES_m_ to ES_w_, respectively. All designs by HT to embedded systems can be fast explored for energy consumption.

**Figure 5:**
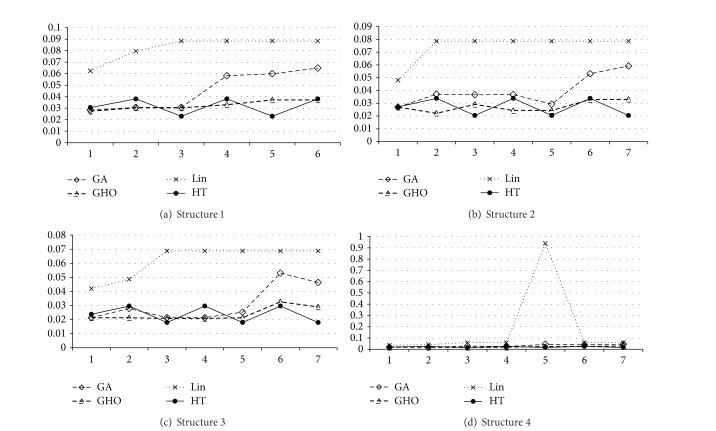
Energy consumption of embedded JPEG encoding system.

To present the efficiency of the proposed HT, we compare HT to* genetic algorithm* (GA) [19], GHO [20], and Lin [21] via JPEG benchmarks. Four structures shown in Figure 4 of energy consumption are used to demonstrate the effects of HT. The energy consumption of each structure is set to 0.1, 0.09, 0.08, and 1 joule, respectively. The structure 1 is shown in Figure 4(a) and the experimental result is depicted in Figure 5(a). The HT gains the energy saving in comparison with GA [19], GHO [20], and Lin [21]. Moreover, the HT improves the energy consumption by 30.00%, 2.38%, and 61.49% on average to GA [19], GHO [20], and Lin [21], respectively.  Figure 5(b) displays the results of structure 2 (Figure 4(b)), in which the HT separately improves the energy consumption by 31.89%, 1.52%, and 63.40% on average to GA [19], GHO [20], and Lin [21].  Figure 5(c) shows the results of structure 3 (Figure 4(c)), in which the HT individually improves the energy consumption by 23.18%, 0.44% and, 61.69% on average to GA [19], GHO [20], and Lin [21].  Figure 5(d) exhibits the results of structure 4 (Figure 4(d)), in which the HT separately improves the energy consumption by 34.28%, 3.93%, and 88.62% on average to GA [19], GHO [20], and Lin [21]. In summary, the HT achieves the energy saving by 29.84%, 2.07%, and 68.80% on average to GA [19], GHO [20], and Lin [21], respectively.

## 5. Conclusion

Energy saving issue is always discussed and concerned in electronic devices. Nowadays, nearly any electronic device either already has existed or will embed computing systems resulting in the applications of embedded systems that are more diverse. It reveals that embedded systems are growing exponentially. While more and more embedded systems are repeated day by day in order to provide various functionalities, the speed of energy consumption is greatly increased.

This study presents* hyperrectangle technology* (HT) to embedded systems target to achieve energy saving. The drift effect on HT facilitates the designer to fast explore energy consumption of embedded systems. The effectiveness of the proposed approach is demonstrated by assessing a JPEG benchmarks. Experimental results demonstrate that the HT achieves the energy saving by 29.84%, 2.07%, and 68.80% on average to GA, GHO, and Lin, respectively. Consequently, this work is valuable for developing energy-saving embedded systems.

## Figures and Tables

**Table 1:** Energy consumption of embedded system with 2 tasks.

Tasks	Energy consumption			
	HC		SC	
	*E*_*d*_^HC^	*E*_*s*_^HW^	*E*_*d*_^SC^	*E*_*s*_^SC^
*v*_1_	5	1	13	9
*v*_2_	6	2	14	10

**Table 2:** Technology of system parameters.

	GA	GHO
Number of chromosomes	100000	100000
Number of populations	500	500
Probability of crossover	1	1
Probability of mutation	1	1

**Table 3:** Measured data of tasks for JPEG encoding system.

Tasks	Energy Consumption			
	HC (10^−6^)		SC (10^−3^)	
	*E*_*d*_^HC^	*E*_*s*_^HC^	*E*_*d*_^SC^	*E*_*s*_^SC^
*v*_0_ (LevelOffset)	0.0180504	0.0119968	0.591286362	0.590508
*v*_1_ (DCT)	1.414320718	0.631151123	181.6586949	138.7103292
*v*_2_ (DCT)	1.414320718	0.631151123	181.6586949	138.7103292
*v*_3_ (DCT)	1.414320718	0.631151123	181.6586949	138.7103292
*v*_4_ (Quant.)	0.629914608	0.123049296	0.93692231	0.934971
*v*_5_ (Quant.)	0.629914608	0.123049296	0.93692231	0.934971
*v*_6_ (Quant.)	0.629914608	0.123049296	0.93692231	0.934971
*v*_7_ (DPCM)	0.000575667	0.000336501	0.040158143	0.040154544
*v*_8_ (ZigZag)	0.02977856	0.003429459	0.561685068	0.5609826
*v*_9_ (DPCM)	0.000575667	0.000336501	0.040158143	0.040154544
*v*_10_ (ZigZag)	0.02977856	0.003429459	0.561685068	0.5609826
*v*_11_ (DPCM)	0.000575667	0.000336501	0.040158143	0.040154544
*v*_12_ (ZigZag)	0.02977856	0.003429459	0.561685068	0.5609826
*v*_13_ (VLC)	0.086972024	0.028369015	0.054136441	0.0541299
*v*_14_ (RLE)	0.046884314	0.023175988	0.838115122	0.836553
*v*_15_ (VLC)	0.093019754	0.030341698	0.054136441	0.0541299
*v*_16_ (RLE)	0.046884314	0.023175988	0.838115122	0.836553
*v*_17_ (VLC)	0.093019754	0.030341698	0.054136441	0.0541299
*v*_18_ (RLE)	0.046884314	0.023175988	0.838115122	0.836553
*v*_19_ (VLC)	0.116959532	0.022825184	0.996227367	0.9940218
*v*_20_ (VLC)	0.116959532	0.022825184	0.976457285	0.9743382
*v*_21_ (VLC)	0.116959532	0.022825184	0.976457285	0.9743382

**Table 4:** Energy consumption of HT.

Embedded systems		HT (ours)			
		Energy dissipation (*J*)			
*v*_1_	*v*_ 22_	Structure 1: Figure 4(a)	Structure 2: Figure 4(b)	Structure 3: Figure 4(c)	Structure 4: Figure 4(d)
ES_a_(0000000000000000000000)		3.9713661	3.544421533	3.117476966	2.69053
ES_b_(0100000000000000000000)		2.680031235	2.391796366	2.103561497	1.81533
ES_c_(0110000000000000000000)		1.38869637	1.239171199	1.089646028	0.94012
ES_d_(0111000000000000000000)		0.097361505	0.086546032	0.075730559	0.06492
ES_e_(0111000000000000000100)		0.088413403	0.078591929	0.068770455	0.05895
ES_f_(0111000000000000000110)		0.079642539	0.070795381	0.061948222	0.05310
ES_g_(0111000000000000000111)		0.070871676	0.062998833	0.05512599	0.04725
ES_h_(0111100000000000000111)		0.0624566	0.055518605	0.04858061	0.04164
ES_i_(0111110000000000000111)		0.054041524	0.048038377	0.04203523	0.03603
ES_j_(0111111000000000000111)		0.045626448	0.040558149	0.03548985	0.03042
ES_k_(0111111000000010000111)		0.038096141	0.033864372	0.029632602	0.02540
ES_l_(0111111000000010100111)		0.030565834	0.027170595	0.023775355	0.02038
ES_m_(0111111000000010101111)		0.023035527	0.020476818	0.017918108	0.01536
ES_n_(1111111000000010101111)		0.017720291	0.015752077	0.013783864	0.01182
ES_o_(1111111010000010101111)		0.012670802	0.011263568	0.009856333	0.00845
ES_p_(1111111010100010101111)		0.007621314	0.006775058	0.005928803	0.00508
ES_q_(1111111010101010101111)		0.002571825	0.002286549	0.002001273	0.00172
ES_r_(1111111010101110101111)		0.002084963	0.001853789	0.001622614	0.00139
ES_s_(1111111010101111101111)		0.001598124	0.001421048	0.001243973	0.00107
ES_t_(1111111010101111111111)		0.001111284	0.000988308	0.000865333	0.00074
ES_u_(1111111110101111111111)		0.000749892	0.000667071	0.00058425	0.00050
ES_v_(1111111111101111111111)		0.000388501	0.000345834	0.000303167	0.00026
ES_w_(1111111111111111111111)		2.711*E* − 05	2.4597*E* − 05	2.20841*E* − 05	1.95711*E* − 05
